# Collision tumors of the sella: coexistence of pituitary adenoma and craniopharyngioma in the sellar region

**DOI:** 10.1186/1477-7819-11-178

**Published:** 2013-08-07

**Authors:** Guishan Jin, Shuyu Hao, Jian Xie, Ruifang Mi, Fusheng Liu

**Affiliations:** 1 Brain Tumor Research Center, Beijing Neurosurgical Institute & Department of Neurosurgery, Beijing Tian Tan Hospital, Capital Medical University, Beijing 100050, China; 2 Department of Neurosurgery, Beijing Tian Tan Hospital, Capital Medical University, Beijing 100050, China

**Keywords:** Collision tumor, Craniopharyngioma, Pituitary adenoma, Sellar region

## Abstract

Collision tumors of the sellar region are relatively uncommon and consist mainly of more than one type of pituitary adenoma or a cyst or cystic tumor. The association of a pituitary adenoma and a craniopharyngioma is particularly rare. This study describes a rare occurrence in which a pituitary adenoma and a craniopharyngioma coexisted in the sellar region. The case involves a 47-year-old woman who underwent transsphenoidal surgery with subtotal tumor resection and reoperation using an interhemispheric transcallosal approach for total microsurgical resection of the tumor because the visual acuity in her left eye had re-deteriorated. Histopathological and immunohistochemical examinations of the excised tissue revealed a pituitary adenoma in the first operation and a craniopharyngioma in the second operation. Retrospective analysis found the coexistence of a pituitary adenoma and a craniopharyngioma, known as a collision tumor. Instead of the transsphenoidal approach, a craniotomy should be performed, to explore the suprasellar region.

## Background

Pituitary adenomas and craniopharyngiomas are two types of common tumors in the sellar or suprasellar areas. Pituitary tumors represent 10% to 15% of all intracranial tumors, with an annual incidence of 0.2 to 2.8 cases per 100,000 persons [1]. Craniopharyngiomas represent 1% to 4% of all primary intracranial neoplasms and occur at a rate of 1.3 per million person years [2]. The coexistence of these two neoplasms in the sellar or suprasellar areas is rare, and both lesions can attain a large size and cause similar signs and symptoms. This similarity makes the diagnosis and treatment of these coexisting tumors difficult. In this report, we present a case in which a pituitary adenoma and a craniopharyngioma were found to coexist. The case involves a 47-year-old woman who underwent transsphenoidal surgery with subtotal pituitary adenoma resection and reoperation using an interhemispheric transcallosal approach for total microsurgical resection of the craniopharyngioma.

## Case presentation

### History and examination

**Figure 1:**
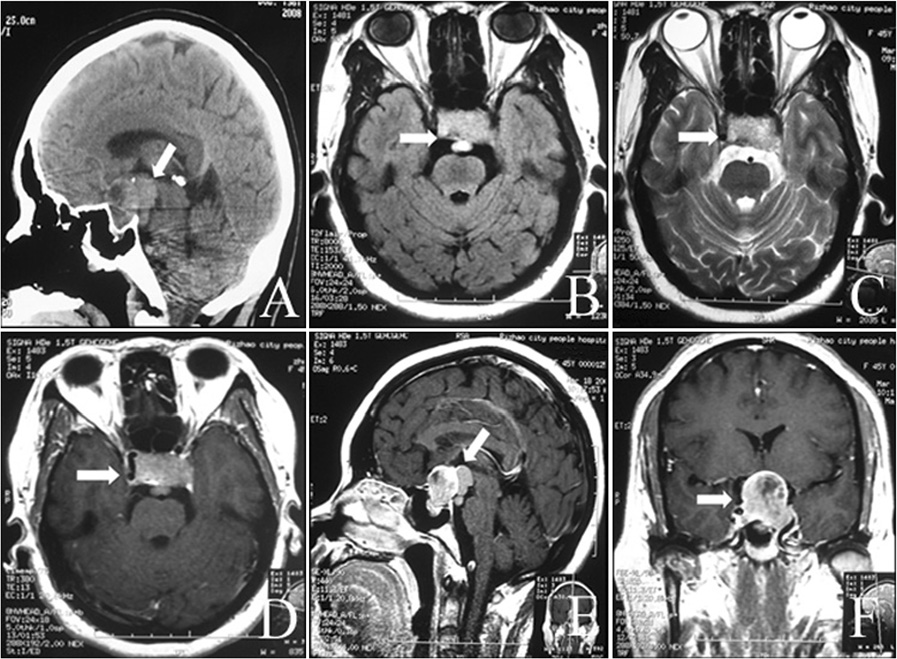
**Preoperative images of computed tomography (CT) and magnetic resonance imaging (MRI) scans. (A)** Sagittal CT showing isodensity or slightly higher density in the sellar and suprasellar areas (arrow indicates the cystic mass in the suprasellar areas). **(B,C)** Transverse MRI showing a short T1-weighted signal in the sellar area and prepontine cistern and a short T2-weighted signal (arrows indicate the mass). **(D-F)** Contrast MRI showing an enhanced mass in the sellar and suprasellar areas with a cystic mass in the prepontine cistern (**D,F**; arrows indicate the mass. **E**; arrow indicates the cystic mass in the prepontine cistern).

A 47-year-old right-handed woman presented with intermittent blurred vision of the left eye and headaches, which she had had for 5 months. She had also suffered from sore roughening or splitting of the palms and arches for 6 months. The patient had been pregnant twice with normal deliveries, and she had not reached menopause at admission. Neurological examination revealed no obvious clinical signs. The patient complained of decreased vision in her left eye. An examination of her visual acuity revealed that her left eye had almost no distant vision. Visual field testing showed that her left eye’s mean sensitivity and mean defect were significantly decreased compared with the normal value. Ophthalmic fundus examination of both eyes did not show any obvious abnormality. The vision of the patient’s right eye was 5/4, and the mean sensitivity and mean defect were decreased but higher than in the left eye. An endocrine evaluation revealed increased levels of prolactin (111.9 ng/ml, reference value: 2 ng/ml to 25 ng/ml) and adrenocorticotropic hormone (ACTH) (116.7 pg/ml, reference value: 11.6 pg/ml to 70.8 pg/ml). Levels of other hormones, including luteinizing hormone, growth hormone, and follicle-stimulating hormone were normal. Other physical examinations revealed that the patient had no other clinical symptoms, neurologic deficits, or other hormonal dysfunction. Biochemical evaluations, including analysis of blood chemistry, electrolyte levels, and urine did not show any obvious abnormality. Computed tomography (CT) and magnetic resonance imaging (MRI) of the patient’s brain revealed an abnormal signal in the sellar and suprasellar areas, owing to the presence of a partial contrasting mass with clear edges (Figure 1). The patient was diagnosed with pituitary adenoma.

### Operation and post-operative course

**Figure 2:**
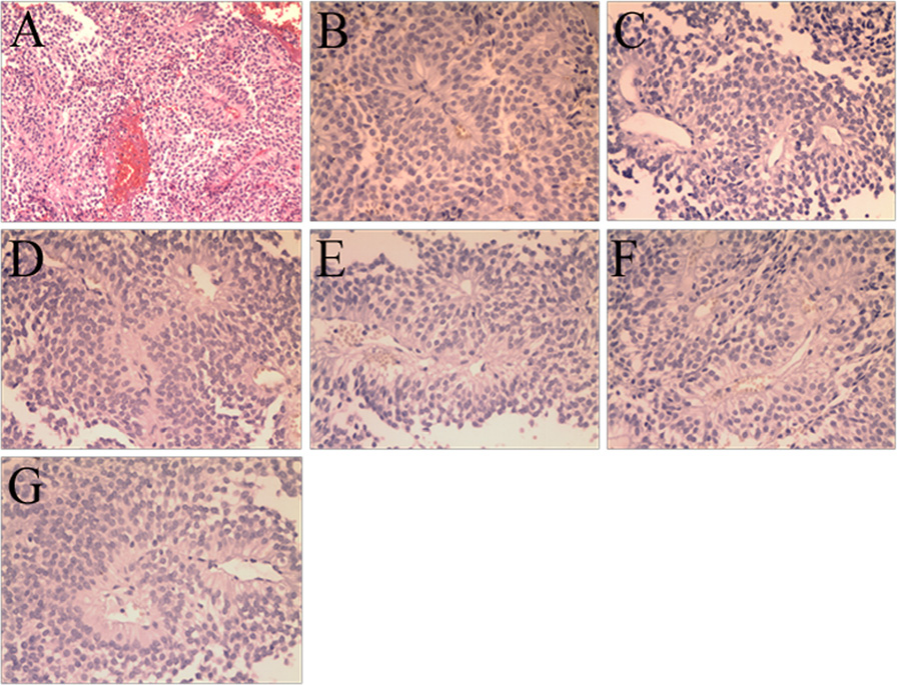
**Photomicrographs of the pathological specimen. (A)** Features of pituitary adenoma (H &E, ×100). Negative immunohistochemical staining for: **(B)** growth hormone, **(C)** prolactin, **(D)** follicle-stimulating hormone, **(E)** thyroid-stimulating hormone, **(F)** luteinizing hormone, **(G)** ACTH.

Transsphenoidal surgery was performed. The tumor was pinkish-gray and soft, and some parts had a rich blood supply with hemorrhage. Subtotal tumor resection was achieved. Staining with H & E revealed a pituitary adenoma consisting of a diffused expansion of cells with pseudo-acinar and pseudo-papillary features (Figure 2A). Immunohistochemical stains for growth hormone, prolactin, follicle-stimulating hormone, thyroid-stimulating hormone, luteinizing hormone, and ACTH were negative, revealing a nonfunctional pituitary adenoma (Figure 2B-G). After surgery, the patient had transient diabetes insipidus and hyponatremia, but she demonstrated fast recovery and her vision improved.

### Second admission and examination

**Figure 3:**
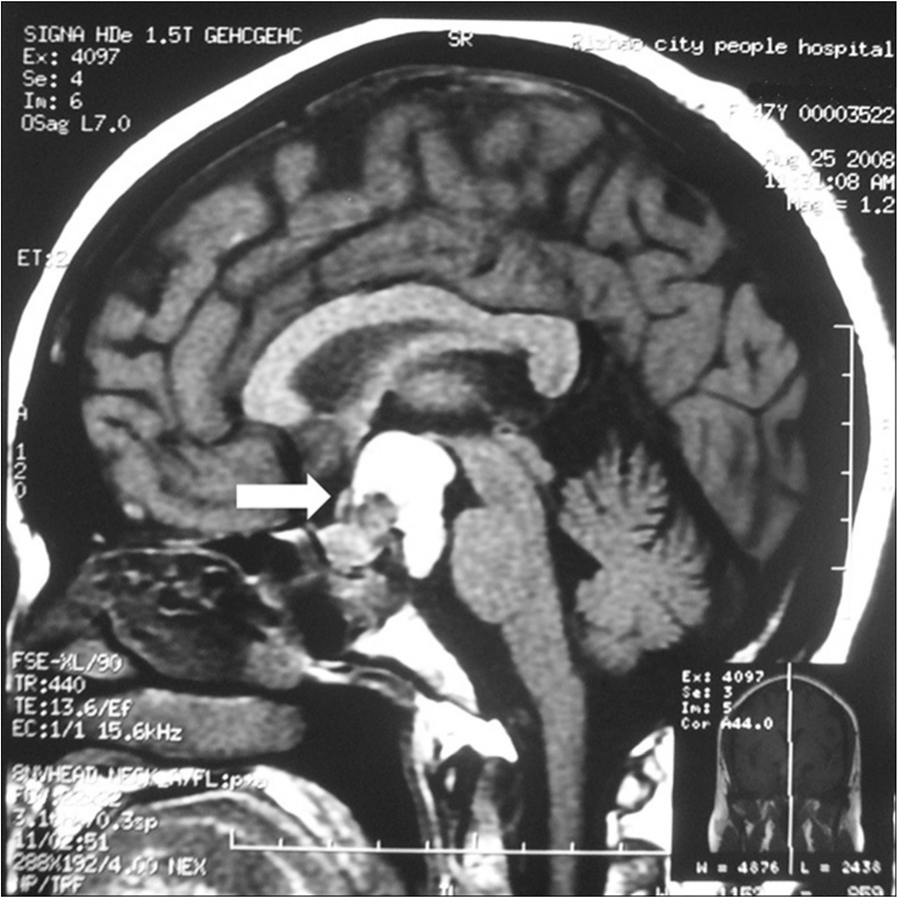
Post-operative MRI scan showing subtotal tumor resection (arrow indicated the enlarged cystic mass).

**Figure 4:**
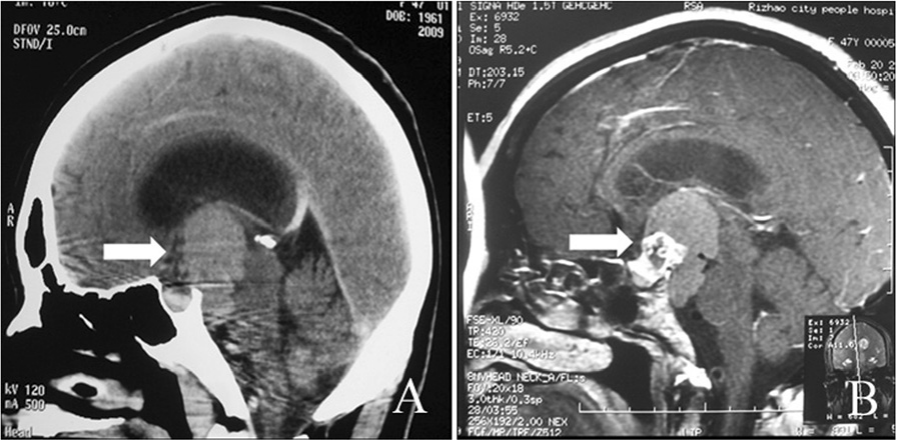
**CT and MRI scans 11 months after the first operation. (A)** Sagittal CT showing the isodensity in the suprasellar area and the prepontine cistern (arrow). **(B)** MRI showing a partial contrasting mass in the suprasellar area (arrow).

After 4 months, a follow-up MRI showed an enlarged region of isodensity in the suprasellar and prepontine areas (Figure 3, arrow point). An enlarged mass corresponding to the ‘cystic lesion’ area was observed in the preoperative image (Figure 1A and E, arrow point). After retrospective analysis, the cystic expansion was believed to be a result of the decompression caused by pituitary tumor resection. The patient was followed up because she had no other clinical symptoms or signs. At 9 months after the initial operation, the patient complained that the visual acuity of her left eye had deteriorated again. Two months later, she came back to our hospital for further examination. Ophthalmologic examination revealed that the status of her left eye was very poor, with almost no visual acuity, and she was not able to maintain visual field detection. Her right eye visual acuity had decreased slightly compared with the previous evaluation (from 5/4 to 5/5), and its visual field was also decreased. Ophthalmic fundus examination of both eyes did not show any obvious abnormality. Endocrinological testing showed normal levels of prolactin, ACTH, follicle-stimulating hormone, luteinizing hormone, growth hormone, and free T4 with only slightly decreased levels of free T3 (1.72 nmol/l, reference value: 2.2 nmol/l to 4.2 nmol/l) and thyroid-stimulating hormone (0.3 μIU/ml, reference value: 0.47 μIU/ml to 4.95 μIU/ml). Sagittal CT and MRI showed an abnormal mixed signal in the suprasellar area and the prepontine cistern, corresponding to a partial contrasting mass with clear edges (Figure 4, arrow point). The patient was diagnosed with recurrent pituitary adenoma.

### Second operation and post-operative course

**Figure 5:**
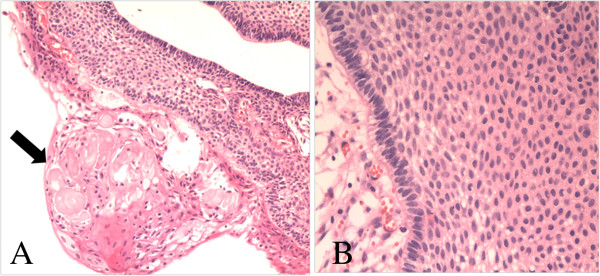
Photomicrographs of the pathological specimen showing the features of craniopharyngioma: (A) wet keratin (arrow, H & E, ×100), (B) multiple layers of squamous epithelium (H & E, ×200).

**Figure 6:**
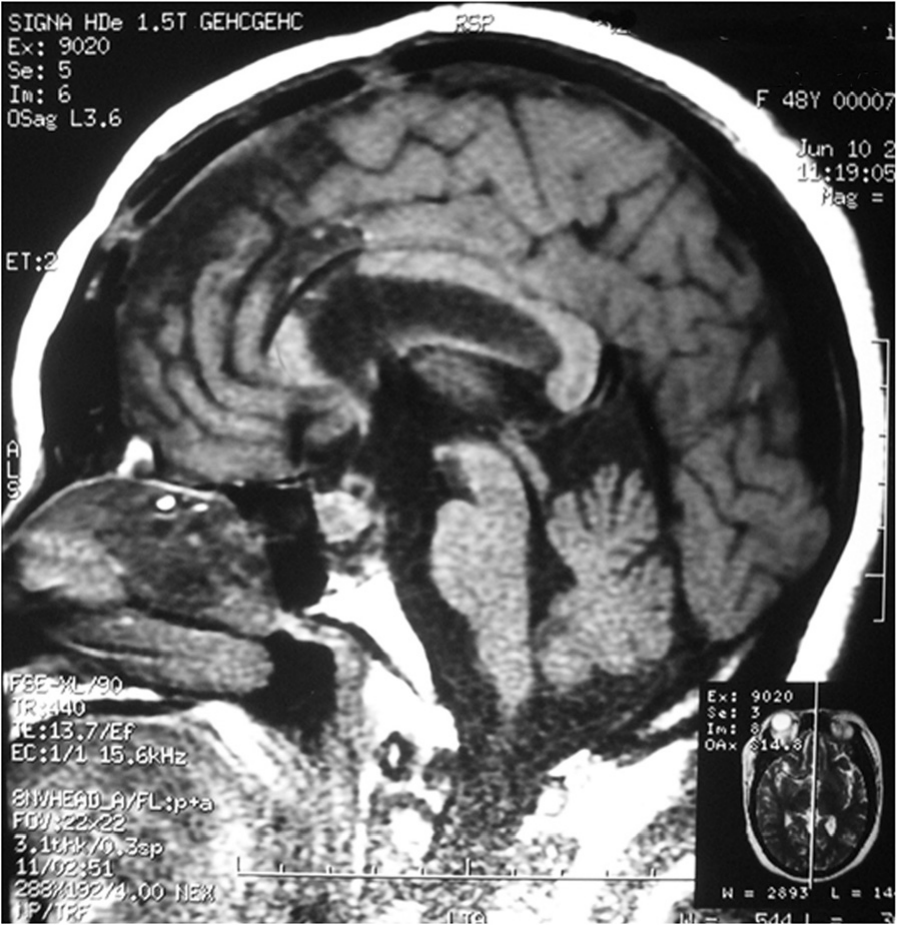
Sagittal T1-weighted MRI scan obtained 3 months after second surgery, revealing total tumor resection.

The patient underwent a right frontal craniotomy using an interhemispheric transcallosal approach for total microsurgical resection of the tumor. The tumor was situated in the suprasellar area and premesencephalon. It was cystic, soft, and yellow-white. Histopathological studies revealed an adamantinomatous craniopharyngioma characterized by squamous epithelium arranged in a trabecular pattern as well as nodules of wet keratin (Figure 5). The post-operative course of the patient was uneventful, with the exception of transient diabetes insipidus and hyponatremia. Endocrinologic testing showed only that levels of free T3 and thyroid-stimulating hormone were slightly lower than normal. The patient’s visual acuity improved again. After 3 months, a follow-up MRI confirmed complete resection of the tumor (Figure 6).

## Discussion

Collision tumors represent two morphologically different tumors that are attached to each other [3]. Although pituitary adenomas and craniopharyngiomas are two of the most common tumors in the sellar or suprasellar areas, the coexistence of a pituitary adenoma and a craniopharyngioma is rare. Only a few cases have been reported; their clinicopathological features are summarized in Table 1[4-16]. Table 1 summarizes 14 cases (including two cases reported in China in Chinese) of tumors found in ten men and four women with ages ranging from 12 to 75. The prolactin type of pituitary adenoma was the most frequent (eight cases), in addition, there were two cases of ACTH (including the present case) and one case of thyroid-stimulating hormone; the remaining cases were the nonfunctional or silent type. The most well-documented cases of craniopharyngioma have been of the adamantinomatous type. The most common clinical features included deteriorating vision and symptoms caused by the abnormal secretion of hormones. These features are similar to those of pituitary adenoma or craniopharyngioma alone. No obvious features were observed in CT and MRI images to identify this coexistence.

**Table 1:** Literature review for the coexistence of pituitary adenoma with craniopharyngioma

**Case**	**Report**	**Age, sex**	**Symptom, duration**	**Hyperprolactinemia**	**1st treatment, pathology**	**Post-operative course**	**Pathologic type of**	**Follow up**
	**year**						**pituitary adenoma**	
1. [4]	1971	29, male	Acromegaly		Right frontal craniotomy, Pituitary adenoma and craniopharyngioma	Diabetes insipidus	Somatotroph	Died 4 days after operation; uncontrolled diabetes insipidus
2. [5]	1981	57, male		Destructive growth pattern	Craniotomy (March 1979) partial removal of craniopharyngioma	2nd surgery : Craniotomy due to regrowth of craniopharyngioma (December 1979)	Prolactin	Died 10 days after operation; cardiac arrest. Chromophobic adenoma (prolactinoma) and chordoma (both post mortem findings)
3. [6]	1986	61, male	Deteriorating vision, 9 months	+	Subfrontal approach, Craniopharyngioma	Visual acuity deteriorated 2 months post-operatively	Prolactin	Autopsy confirmed pituitary adenoma
						Died from cardiac arrest.		
4. [7]	1986	32, female	Amenorrhea, lactation, acromegaly, 3 months	+	Transsphenoidal, Pituitary adenoma	1st surgery: headache and visual acuity deteriorated 1 month post-operatively	Prolactin and growth hormone	Not known
						2nd surgery: right frontal craniotomy for craniopharyngioma		
5. [8]	1987	47, male	Deteriorating vision	+	Transsphenoidal, Pituitary adenoma	1st surgery: visual acuity deteriorated 1 month post-operatively 2nd surgery: interhemispheric approach without pathological changes	Prolactin	Not known
						3rd surgery: bifrontal craniotomy for craniopharyngioma		
6. [9]	1987	36, male	Deteriorating vision, 18 months		Transsphenoidal Pituitary adenoma	1st surgery: headache and visual acuity deteriorated 2 months post-operatively	Nonfunctional adenoma	Not known
						2nd surgery: craniotomy for craniopharyngioma		
7. [10]	1988	62, female	Personality change, 2 months	+	Right frontoparietal parasagittal craniotomy and radiotherapy, Craniopharyngioma	Lethargy, ataxia, incontinence, polyuria and polydipsia 12 months post-operatively	Prolactin	Autopsy confirmed lactotroph hyperplasia and microprolactinoma
						Died from pulmonary embolism		
8. [11]	2008	29, male	Atrial fibrillation, 24 months	−	Transsphenoidal Composite, pituitary adenoma and craniopharyngioma	Not known	Thyroid-stimulating hormone	Not known
9. [12]	2008	50, male	Headache, difficulty sleeping, decreased libido	+	Transsphenoidal, Pituitary adenoma and craniopharyngioma	Hypogonadal	Gonadotrophic hormone	No recurrence in 4 years
10. [13]	2009	59, male	Progressive vision loss		Subtotal transcranial resection	Transient diabetes insipidus	Gonadotrophic hormone	Not known
11. [14]	2009	12, male	Partial hypopituitarism	+	Right frontal craniotomy, Composite craniopharyngioma and pituitary adenoma	Uneventful	Silent pituitary adenoma subtype 3	MRI performed 8 months post-operatively; 10 months after operation no tumor recurrence
12. [15]	2009	47, male	Headache and vision loss, years	−	Transsphenoidal, Composite pituitary adenoma and craniopharyngioma	Uneventful	Nonfunctional adenoma	No recurrence in 1 year
13. [16]	2013	75, female	Diplopia	+	Transsphenoidal, Composite pituitary adenoma and craniopharyngioma	Uneventful	Silent type 2, ACTH	No recurrence in 10 months
14. Present case	2009	47, female	Deteriorating vision, 5 months	+	Transsphenoidal, Pituitary adenoma	1st surgery: visual acuity deteriorated 9 months post-operatively	prolactin and ACTH	No recurrence in 2 years
						2nd surgery: interhemispheric transcallosal approach		

*ACTH* adrenocorticotropic hormone.

Given the similarities of the clinical and imaging features to those of pituitary adenomas, a preoperative diagnosis of a dual pathological condition of the sella is highly difficult. A definitive diagnosis of a collision sellar lesion is usually based on histological studies. In this case, preoperative MRI showed two abnormal masses attached to each other with different signal intensities (Figure 1A,E). The calcification on the CT imaging in Figure 1A was ignored at first, and the preoperative imaging findings were considered to indicate a pituitary adenoma with cystic changes. The cystic mass was expected to shrink naturally after transsphenoidal surgery of the pituitary adenoma, but instead it increased in size after the surgery. The cystic mass was still not given sufficient attention, and it was believed that the cystic dilatation had been caused by the decreased pressure after removal of the pituitary adenoma. It was only when the patient’s visual acuity in her left eye deteriorated and the imaging was repeated that the tumor recurrence was identified. A review of the histological diagnoses of this case, namely, a pituitary adenoma in the first operation and a craniopharyngioma in the second operation, revealed that ‘a pituitary adenoma with cystic changes’ was in fact coexisting pituitary adenoma and craniopharyngioma and that ‘the cystic changes’ were features of a craniopharyngioma lesion. Although the transsphenoidal approach is the first choice for the resection of most pituitary adenomas, craniotomy should be performed to explore the suprasellar region for coexisting tumors. If the cystic part can be identified as a craniopharyngioma during the first procedure, a craniotomy should be performed to explore the suprasellar region, rather than the transsphenoidal approach, and a second surgery might thereby be avoided. After the second operation, the tumor was completely removed surgically using the interhemispheric transcallosal approach. During a follow-up period of 3 months, the patient was alive and well, with no evidence of disease progression. MRI showed total tumor resection with post-surgical changes (Figure 6).

The tumorigenesis of the coexisting pituitary adenoma and craniopharyngioma is unclear. Yoshida *et al*. [11] reported a case of pituitary adenoma intermingled with adamantinomatous craniopharyngioma-like components in a young man, but an intermediate morphological phenotype was not found between the two types of tumor. Gokden and Mrak [15] reported a nonfunctioning pituitary adenoma with an intermingled craniopharyngioma component that did not form two distinct lesions. In their study, a histological delineation was also absent, although multiple foci corresponding to the transition from an ordinary pituitary adenoma to an adenoma with squamoid and then adamantinomatous areas were described by pathological examination. Moshkin *et al*. [14] reported a collision tumor, and histological analysis revealed it to be an adamantinomatous craniopharyngioma. However, electron microscopy showed adenoma cells with the ultrastructural features of a silent pituitary adenoma subtype 3. Aside from these three cases, in which the pituitary adenoma and craniopharyngioma components were admixed, additional cases in which the two components are distinct have been reported. Our study describes another case with the association of a pituitary adenoma and a craniopharyngioma, in which the two components are separated. In general, a neoplasm with dual internal phenotypes is can be explained using metaplastic mechanisms, but the histogenesis of the aforementioned collision tumors is still uncertain. Among the cases of collision tumors listed in Table 1, eight cases had hyperprolactinemia. With respect to the tumorigenesis of these types of concurrent tumor, Cusimano *et al*. [10] postulated that the loss of the inhibitory hypothalamic dopaminergic input due to pituitary stalk compression by craniopharyngioma is intimately associated with the pathogenesis of lactotroph hyperplasia and prolactinoma. In updated cases, reports on the pathological types of pituitary adenomas other than prolactinoma are available. However, the malfunction of hypothalamic dopaminergic input caused by craniopharyngioma does not explain the formation of nonfunctional or ACTH adenoma. A determination of the mechanisms responsible for the collision coexistence of the two tumor types requires further study.

## Conclusions

The concurrence of pituitary adenoma and craniopharyngioma is rare and represents a serious problem in clinical and imaging diagnoses. If we had been aware of the possibility of the concurrence (it is completely possible to make a correct diagnosis based on pathology and imaging characteristics), we would have avoided the second operation resulting from the misdiagnosis. For a patient with a pituitary adenoma and craniopharyngioma collision tumor, a craniotomy should be performed to explore the suprasellar region, rather than a transsphenoidal approach.

## Consent

Written informed consent was obtained from the patient for publication of this case report and any accompanying images. A copy of the written consent is available for review by the editor-in-chief of this journal.

## Abbreviations

ACTH: Adrenocorticotropic hormone; CT: Computed tomography; H & E: Hematoxylin and eosin; MRI: Magnetic resonance imaging.

## Competing interests

The authors declare that they have no competing interests.

## Authors’ contributions

GJ and SH reviewed the literature and drafted the manuscript. FL was clinically responsible for the patient’s care and revised the manuscript. JX and RM were responsible for the pathology and radiological images. All authors read and approved the final manuscript.
